# Solvent-Induced Raynaud’s Phenomenon

**DOI:** 10.7759/cureus.45004

**Published:** 2023-09-11

**Authors:** Helena Rodrigues, Catarina Reigota, Carolina Teles, Sónia Moreira, Lèlita Santos

**Affiliations:** 1 Internal Medicine, Centro Hospitalar e Universitário de Coimbra, Coimbra, PRT

**Keywords:** secondary raynaud’s phenomenon, iloprost, occupational risk, solvent exposure, raynaud’s phenomenon

## Abstract

Raynaud’s phenomenon (RP) is a vasospastic disorder characterized by an exaggerated vasoconstrictive response to cold or emotional stress. It can be classified as primary (PRP) or secondary (SRP) depending on its association with an underlying condition. We present a case of a young female with severe RP, with trophic changes and abnormal capillaroscopy. After a detailed investigation, a diagnosis of secondary RP due to solvent exposure was made. The patient was treated with calcium channel blocker in low doses, due to hypotension, without improvement of symptoms. Vitamin C and pentoxifylline were added with an unsatisfactory response. Given the progressive worsening of RP and the appearance of trophic lesions, the patient’s treatment was reviewed, and continuous intravenous iloprost infusion through an elastomeric pump was started. This resulted in significant symptom improvement and normalization on the capillaroscopic examination.

## Introduction

Raynaud’s phenomenon (RP) was first described in the 19th century by Maurice Raynaud as a vasospastic disorder of the digital vessels triggered by exposure to cold or stress [1]. These episodes of digital ischemia are characterized by a classic triphasic color change from white/pallor to blue and/or to red, corresponding to ischemia, venostasis and deoxygenation, and reactive hyperemia, respectively [2,3]. There may also be trophic changes limited to the skin and uncomfortable sensory symptoms (numbness and paresthesia) of the extremities [3,4].

The pathophysiology of RP is complex, and many different mechanisms have been implicated, such as vascular, neural, and intravascular abnormalities [5]. Estrogen has also been implicated in its pathophysiology. For this reason, RP is 3-4 times more common in females than in males [5-7].

RP can be classified as primary or secondary. Primary RP (PRP) is an idiopathic and benign condition, tends to be milder, and has an earlier age of onset. The prevalence of PRP varies according to diagnostic criteria and geographic localization, ranging between 5% and 20% in the general population [2,5,8].

Secondary RP (SRP) can be associated with autoimmune diseases, arterial or neurological disorders of the upper extremities, environmental or occupational exposures (vibration, microtrauma, and solvents), drug side effects (more importantly beta-blockers), oncological and hematological conditions (such as dysglobulinemia, myeloproliferative disorders, and paraneoplastic syndromes), and hypothyroidism. SRP can evolve into digital ulceration, scarring, and gangrene [2,5,9].

The pathophysiology underlying solvent-induced RP is not clear; however, several pathways are proposed. It is speculated that solvents could induce endothelial injury through different pathways, namely, by (1) increasing levels of vasoconstrictors and decreasing levels of vasodilators, (2) inducing cellular damage by binding to nucleic acids and proteins, (3) reducing humoral and cell-mediated immune responses, and (4) stimulating the production of fibrogenic proteins and growth factors such as interleukin 1 (IL-1), platelet-derived growth factor, transforming growth factor (TGF)-ß, and fibronectin [5,10-12].

Treatment for RP depends on the underlying cause and severity and includes behavioral changes and the use of calcium channel blockers such as amlodipine and nifedipine, phosphodiesterase type 5 (PDE-5) inhibitors, and prostanoids such as iloprost [1,2].

This report describes the case of a patient with severe RP, caused by occupational exposure, effectively managed with continuous intravenous iloprost through an elastomeric pump.

## Case presentation

A female in her mid-20s was admitted to the Internal Medicine Department for clinical investigation of bilateral Raynaud’s phenomenon and skin changes that had begun a year before. She complained of episodes of change in the color of the digits of both hands ranging between pallor, cyanosis, and redness, with mild pain, numbness, and swelling. These episodes progressively worsened and were, at the time of the first evaluation, not related to low temperature. She complained of swelling and skin changes of the fingers with small desquamative lesions, xerostomia, xerophthalmia, photosensitivity, generalized muscle weakness, and mechanical pain in several joints (interphalangeal, metacarpophalangeal, and neck and back pain). The patient denied joint swelling and stiffness, fever, adenopathy, hair loss, nail pitting, rash, oral or genital ulcers, skin thickening, dysphagia, shortness of breath, chest pain, or cough.

She worked in a hairdresser salon as a nail stylist and designer and denied using personal protection equipment (protective gloves or facial mask) at work and denied adequate ventilation. Skin contact and inhalation were admitted by the patient, but the specific amounts of chemicals were not quantifiable. She had daily contact with chemical products such as xylene and acetone, working more than eight hours per day and resting only on Sunday.

She lived in a rural environment referring to recurrent contact with chickens, pigs, and rabbits. Her medical history included anxiety disorder, allergic rhinitis, hyperhidrosis, recurrent urinary tract infections, and an episode of septic arthritis of the right shoulder in childhood. She was not taking any birth control pills. She drank alcohol occasionally and denied smoking or drug use. She had a family history of cardiovascular disease and had a cousin with Crohn’s disease.

Previous treatments for her RP included behavioral modification, amlodipine, vitamin C, and pentoxifylline. Amlodipine was only tolerated up to 5 mg/day. At the time of admission, she was medicated with amlodipine, aspirin, and pentoxifylline. Despite this treatment, her Raynaud's phenomenon was worsening, and she was therefore admitted to the Internal Medicine Department to start treatment with iloprost.

**Figure 1:**
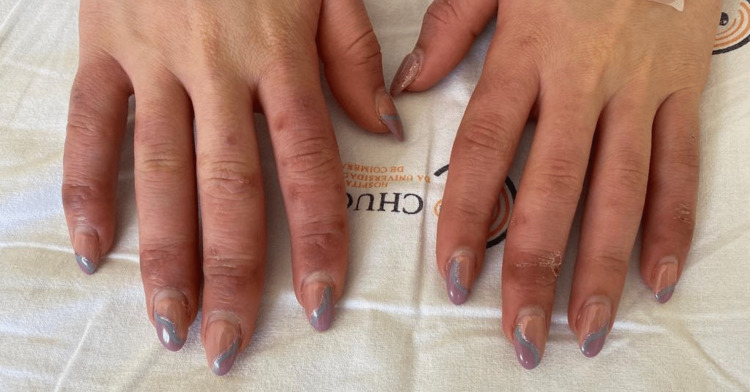
Skin lesions and trophic changes before treatment.

Upon our initial assessment, she exhibited hyperhidrosis in her hands and had inflammatory signs, limited to the skin, over the interphalangeal joints of both hands, with small skin erosions and trophic alterations in the fingers (Figure 1). There was no palpable thickening, pitting, ulcers, or scars. She had strong arterial pulses bilaterally and had no motor or sensitive changes. The remaining rheumatological assessment showed trapezius muscle contracture bilaterally, pain with the abduction of both arms above 90°, and pain with active and passive mobilization of the tibiotarsal joint, without inflammatory signs.

**Figure 2:**
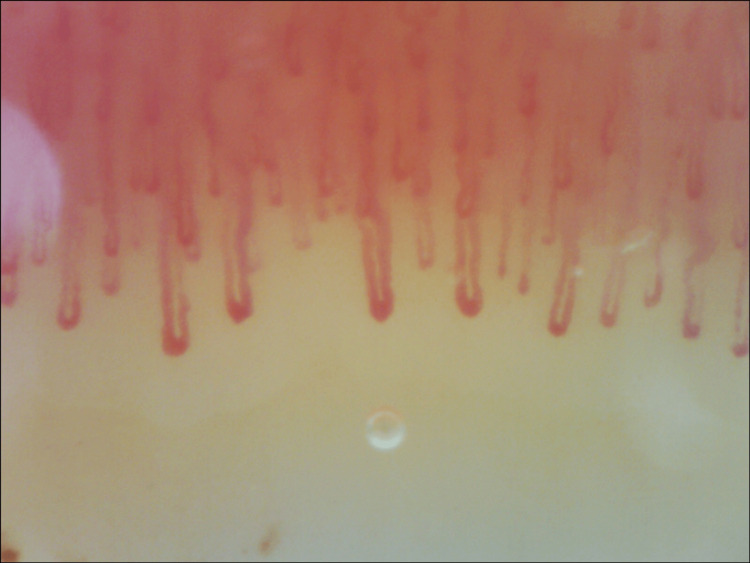
Nailfold capillaroscopy before treatment.

To investigate a possible underlying cause for this Raynaud’s phenomenon, further tests were requested. Complete blood cell count and biochemistry with muscle enzymes were normal. There were no vitamin or iron deficiencies. Thyroid hormones were within normal range. Laboratory tests were negative for antinuclear antibodies, as well as anti-double-stranded DNA (anti-dsDNA), anti-centromere, anti-SCL-70, anti-RNA polymerase III, anti-ribonucleoprotein (anti-RNP), anti-La/Sjogren’s syndrome B (SSB), and anti-Sjogren’s syndrome A (SSA)/Ro antibodies. Immunoglobulins and complement levels were within normal limits. Serological tests were negative for hepatitis virus B and C, human immunodeficiency virus (HIV), herpes simplex virus (HSV), Epstein-Barr virus (EBV), *Chlamydia trachomatis*, *Borrelia*, *Rickettsia*, and *Coxiella*. Interferon-gamma release assay (IGRA) was also negative. Chest X-ray showed no abnormalities, lung function tests were normal, and chest, abdominal, and pelvic computed tomography showed no malignancy or other pathological findings that could justify a paraneoplastic RP. Magnetic resonance excluded sacroiliitis, and no inflammatory activity was identified in the PET scan. Subclavian Doppler ultrasound showed normal filling of the lumen, and the Doppler study was normal. Nailfold capillaroscopy (Figure 2) revealed non-specific changes bilaterally, such as capillary enlargement (34-49 microns), without megacapillaries, rare capillary tortuosity, a few small traumatic hemorrhages, and pericapillary edema, with normal density (10 capillaries/mm^2^) and no avascular areas. These changes were compatible with a non-scleroderma-like pattern.

During the evaluation of the patient, some differential diagnoses were considered. Due to the age and sex of our patient, the variety of symptoms presented (arthralgia, xerostomia, xerophthalmia, photosensitivity, and fatigue), and the abnormalities on nailfold capillaroscopy, the hypothesis of being a rheumatological disease was first considered and therefore excluded. Obstructive arterial disease (such as atherosclerosis and arterial embolism) was excluded by physical examination and Doppler study. Malignant disease with paraneoplastic acral vascular syndrome was the least likely hypothesis and was excluded by chest, abdominal, and pelvic computed tomography. At the end of our study, the most likely diagnosis was Raynaud’s phenomenon secondary to solvent exposure (xylene and acetone).

While an inpatient, treatment with intravenous iloprost (250 micrograms of iloprost diluted in saline) using an elastomeric pump was started. The pump was connected to a peripheral catheter, which was placed in a medium-sized vein, in the forearm. The treatment was kept for five consecutive days. Iloprost infusion through an elastomeric pump was repeated monthly over six months, as an outpatient. The treatment was generally very well tolerated, reporting occasionally mild headaches. Nausea, flushing, or hypotension were not reported.

The patient was also advised to remove the gel nails and discontinue contact with solvents, considering the association between her exposure and the development of Raynaud’s phenomenon.

**Figure 3:**
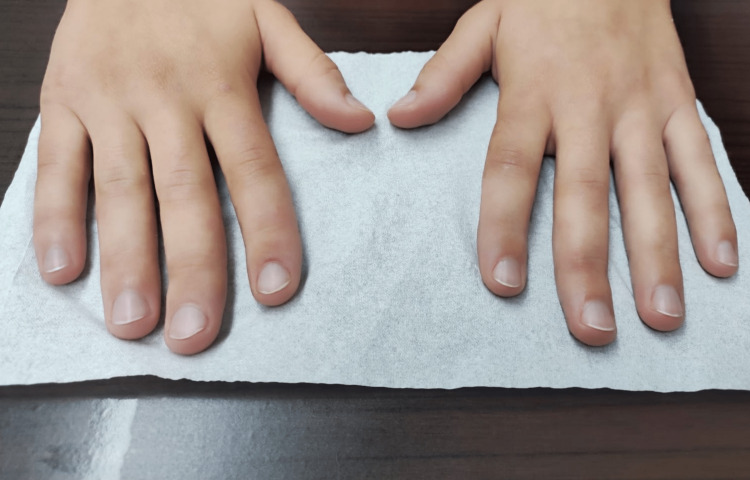
Patient’s hands six months after treatment.

RP resolved after six months and did not recur during the one-and-a-half-year follow-up period. The trophic lesions had healed completely, and the patient was asymptomatic (Figure 3).

**Figure 4:**
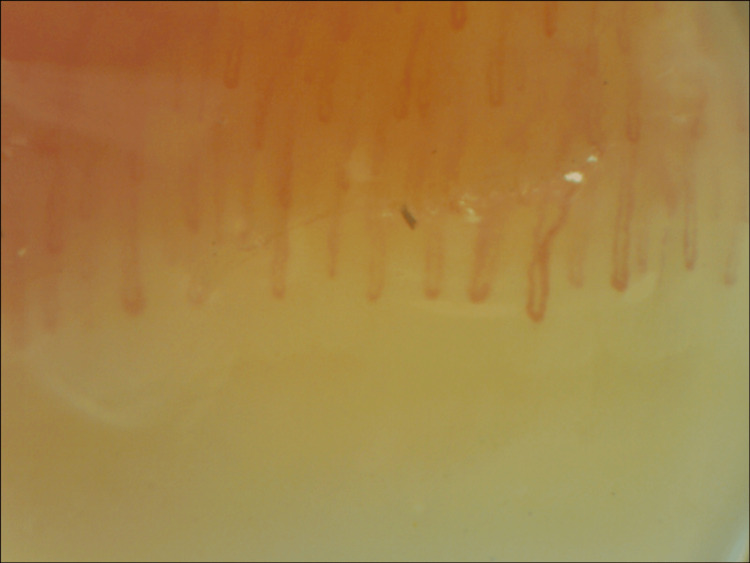
Nailfold capillaroscopy after treatment.

One-year follow-up capillaroscopy (Figure 4) showed significant improvement in capillary enlargement and less tortuosity, with most capillaries being normal in shape and size.

The patient refrained from solvent exposure by consistently using personal protective equipment and discontinuing the practice of painting her own nails. Currently, she shows no symptoms of RP and is not taking any medication.

## Discussion

We found this case challenging due to the multitude of symptoms that the patient developed since the beginning of her RP. Despite her young age, the unspecific changes in capillaroscopy, and the trophic changes, a complete study of secondary causes was conducted, allowing us to diagnose a secondary RP by occupational exposure to solvents. The association between exposure to certain solvents (particularly xylene and toluene) and the development of severe RP had been previously described [13-15]. Our patient had been working with nail polishers containing xylene and acetone for approximately eight years before the onset of RP. The severity of her RP can also be explained by the prolonged exposure to the solvents, as seen in the study of Purdie et al. [13].

Several studies have explored the effects of estrogen on vascular function and endothelial function, unrevealing its potential influence on Raynaud’s phenomenon. Since this patient is premenopausal, estrogens may also contribute to the exacerbation of the condition [7,16].

It is crucial to note that the patient is currently under regular medical care and not taking any medication. Ongoing monitoring and evaluation are essential to assess long-term implications and to rule out any evolving autoimmune disease. This ensures that appropriate care is provided throughout her treatment journey.

The present case report also illustrated the successful use of iloprost in a patient with severe RP that was intolerant to the first lines of therapy. The European League Against Rheumatism recommends the use of intravenous iloprost for the treatment of systemic sclerosis (SSc)-related digital vasculopathy, as it reduces the frequency and severity of SSc-RP attacks and heals active digital ulcers [17]. This treatment also significantly reduced the severity of RP attacks, including numbness, pain, and color changes, and improved trophic changes and capillaroscopy patterns in our patient with solvent-induced RP.

## Conclusions

In conclusion, the management of severe RP in patients with low tolerance to high doses of calcium channel blockers presents a notable challenge. The use of intravenous iloprost with an elastomeric pump treatment allows the administration of home care, an approach that reduces the burden of disease.

Furthermore, the investigation of secondary RP remains a critical aspect of comprehensive RP management. Occupational exposures, among other factors, warrant careful consideration, as they can significantly contribute to the development and exacerbation of RP. Incorporating a thorough understanding of these underlying causes into the management strategy can help optimize treatment outcomes and enhance the overall well-being of individuals affected by Raynaud’s phenomenon.
